# Monoclonal antibodies against CGRP (R): non-responders and switchers: real world data from an austrian case series

**DOI:** 10.1186/s12883-023-03203-9

**Published:** 2023-04-28

**Authors:** Katharina Kaltseis, Vera Filippi, Florian Frank, Christine Eckhardt, Alois Schiefecker, Gregor Broessner

**Affiliations:** Department of Neurology, Medical University of Innsbruck, Innsbruck, Austria

**Keywords:** Migraine, Monoclonal antibodies, CGRP, Non-responders, Switchers, Real-world-data

## Abstract

**Objective:**

Assessement of the responder and non-responder rate to consecutive monoclonal CGRP-antibody (CGRP-mAb) treatment, the presence of side effects, analysis of predictors of response and loss-of-effectiveness evaluation over time.

**Methods:**

We conducted a retrospective analysis including 171 patients with episodic (EM) or chronic migraine (CM), who received one, two or three different CGRP-mAbs. Non-response was defined as ≤ 50% reduction of monthly migraine days (MMDs) in EM and ≤ 30% reduction of MMDs in CM after 3 months of treatment.

**Results:**

123 (71.9%) responded to the first mAb. Side effects led to treatment discontinuation in 9 (5.3%) patients. Of the 26 patients who did not respond to the first mAb or experienced a loss of efficacy over time, 11 (42.3%) responded to the second and two (28.6%) of 7 to the third monoclonal antibody. Poor response to therapy was associated with a higher monthly migraine frequency (p = 0.028), a higher number of prior preventive migraine therapies (p = 0.011) and medication overuse (p = 0.022).

**Conclusion:**

Our findings support mAb-class switch in non-responders or in patients experiencing a loss of effectiveness. The use of a third CGRP-mAb could be beneficial for some patients.

## Introduction

With the discovery of the neuropeptide calcitonin gene-related peptide (CGRP) in 1983 [1] and the confirmation of its importance in pain perception [2] as well as in the pathophysiology of migraine [3], the understanding and treatment of this primary headache disorder has been turned upside down ever since. The development of CGRP-ligand (eptinezumab, galcanezumab, fremanezumab) and CGRP-receptor (CGRP(R), erenumab) antibodies heralded a new era – for the first time, preventive treatment specifically addressing CGRP in migraine are available.

Clinical trials of all monoclonal antibodies (mAbs) showed excellent efficacy with a 50%- response rate in 39-62% of patients with episodic migraine (EM) [4–7] and 27-57% for chronic migraine (CM) [8–11], respectively. Recently published real-world data on the use of these substances in episodic migraine [12] as well as in a difficult-to-treat considered patient group experiencing chronic migraine [13] and medication-overuse-headache (MOH) [14, 15] affirmed their potential. Literature suggests that CGRP-mAbs, along with their favorable safety and tolerability profile, might be superior compared to previously established preventive migraine therapies such as beta-blockers, antiepileptics, calcium-channel blockers, or tricyclic antidepressants: A meta-analysis comparing topiramate to CGRP-antibodies in EM confirmed the excellent tolerability of monoclonal antibodies despite a comparable reduction of migraine days [16]. The comparison of CGRP-antibodies, topiramate, and onabotulinumtoxinA in EM and CM revealed the highest effect size regarding 50% reduction of headache days, but also the greatest drop-out rate in the individuals treated with topiramate [17]. However, these results are based upon clinical trials including pre-selected patient population and not on “real-world data”. Only recently the first randomized, double-blind, controlled head-to-head study by Reuter et al. was published and demonstrated a significantly higher 50%-response rate and tolerability of erenumab compared to topiramate in the prevention of migraine [18].

Despite these promising results, approximately 15–25% of migraineurs do not respond to CGRP antibodies irrespective of CGRP-ligand or -receptor blockade [14, 19]. It has been demonstrated that some patients benefit from switching mAb classes [20, 21], nevertheless few patients must be classified as non-responders. Currently, the reason for this phenomenon is still elusive – though suggesting that the CGRP pathway might only partly explain migraine pathophysiology.

The aims of the current study were to assess (i) the responder and non-responder rate to consecutive CGRP-mAb treatment, (ii) the presence of side effects as well as (iii) the loss of efficacy in a subset of patients with EM and CM receiving up to 3 different CGRP-mAbs.

## Methods

This retrospective, real-world case series was conducted at the tertiary headache center of the Medical University of Innsbruck, including 196 patients with EM and CM who have received their first dose of a monoclonal CGRP(R)-mAb (erenumab 70 mg/month or 140 mg/month) or a CGRP-ligand antibody (galcanezumab 120 mg/month with a loading dose of 240 mg or fremanezumab 225 mg/month or 675 mg/quarterly) between April 2018 and December 2021. During the study period, eptinezumab was not approved for the preventive treatment of migraine in adults in the European Union and was therefore not included in the registry.

Data on migraine headache frequency, previous therapeutic approaches, comorbidities, and the use of acute medication was collected during outpatient visits using a structured headache interview, medical records, and headache diaries. Follow-up visits were scheduled 3 to 12 months after treatment initiation with a mAb to evaluate the response to therapy. Migraine headache frequency in the month prior to the start of a mAb was used as baseline monthly migraine days (MMDs).

Headaches were classified in accordance with the latest International Classification of Headache Disorders, 3rd edition (ICHD-3) [22]. Patients with > 1 and < 15 headache days/month were diagnosed with EM and patients with ≥ 15 headache days/month were classified as CM [22]. In Austria, treatment with a CGRP-mAb is reimbursed in patients (1) aged ≥ 18 years, (2) with ≥ 4 migraine days per month, (3) no therapeutic response, the occurrence of side effects or contraindications of > 3 preventive migraine medications such as beta-blockers, antiepileptics, calcium channel blockers, onabotulinumtoxinA, tricyclic antidepressants.

Additional headache diagnoses (referred to as “other” in Table 1) included tension-type headache (TTH), trigeminal-autonomic cephalalgias (TACs) and secondary headaches like headache attributed to traumatic injury to the head or headache attributed to increased cerebrospinal pressure. Medication overuse headache (MOH) was classified according to the ICHD-3 as regular use of one or more non-opioid analgesics (NSAIDs, paracetamol or acetylsalicylic acid) on 15 or more days/month or regular use of triptans, ergotamines, opioids or combination analgesics on 10 or more days/month for headache treatment in the last 3 months [22]. Inpatient and/or outpatient withdrawal due to medication overuse was recorded.

Prior preventive migraine medications included beta-blockers (metoprolol, propranolol), angiotensin receptor blocker (candesartan), anti-epileptic drugs (topiramate, valproic acid), calcium channel blocker (flunarizine), tricyclic antidepressant (amitriptyline), selective serotonin and norepinephrine reuptake inhibitor antidepressants (venlafaxine), and tanacetum parthenium. Preventive therapies, which were used by less than 5 patients were included in the “Other” group (gabapentin, duloxetine, pregabalin, amalium, citalopram, zonisamide, mirtazapine, tizanidine). OnabotulinumtoxinA was only used in patients with CM and administered according to the “PREEMPT” injection protocol [23]. The reason for discontinuing the treatment was assessed during structured headache interviews and via medical records and included side effects (yes/no), loss of efficacy, non-response, and other reasons. Loss of efficacy was defined as an initial response, but with a subsequent increase in migraine frequency back to or beyond the baseline level.

As migraine is associated with a wide range of psychiatric disorders [24], we included the prevalence of depression, anxiety and/or panic disorders as well as eating disorders in our analysis. Disease duration was calculated for each patient from the first migraine attack until the first treatment with a monoclonal antibody.

Patients were considered as non-responders, if they did not show a therapeutic response, defined as ≥ 50% reduction of monthly migraine days (MMDs) in EM and ≥ 30% reduction of MMDs in CM after an adequate treatment duration of at least 3 months. Thereby, we followed the latest guidelines of the European Headache Federation (EHF) suggesting an evaluation of efficacy after 3 months of consecutive treatment with a monoclonal antibody targeting the CGRP pathway [25]. Therapeutic response was evaluated during the follow-up visits scheduled between 3 and 12 months after treatment initiation. Lack/Loss of efficacy and side effects causing a switch to another mAb were documented. Loss of efficacy was defined as an initial response, but with a subsequent increase in migraine frequency back to or beyond the baseline level. If the mAb was discontinued for other reasons (wishing to conceive, reimbursement issues), the patients were included in the category “other”. Patients switching antibody treatment, regardless of whether non-response, loss of efficacy, or adverse events led to treatment discontinuation, were advised to pause CGRP-mAb treatment at least 2 to 3 months before starting a new mAb. If the patient received a CGRP-receptor antibody as the first drug, they were treated with a CGRP-ligand antibody as second therapeutic attempt and vice versa. Demographic and clinical characteristics were collected from the electronic patient documentation database. As a detailed history was taken during the initial consultation and follow-up visits to the headache outpatient clinic, there was no missing data for the selected variables, except for those patients who requested further care in general practice. Due to the lack of follow-up data, these patients were not included in the final analysis.

### Statistics

Statistical analyses were performed using SPSS Statistics (version 27.0; IBM Corporation, Armonk, NY, US). Normal distribution of data was assessed with the Kolmogorov-Smirnoff Test. Data are given as mean ± standard deviations (SD) for normally distributed data and medians and interquartile ranges [IQR] for non-normally distributed data. Continuous variables, categorical variables are presented as percentages. The Student´s t-test or Mann-Whitney-U test were applied as appropriate. We ran a multinomial logistic regression model adjusting for: gender (male, female), age, non-daily migraine (yes/no), psychiatric disorder (yes/no), number of previously used prophylactic medications, mean monthly migraine days, medication-overuse (yes/no), withdrawal (yes/no). The level of significance was set at p < 0.05. As this was a retrospective data analysis, a sample size calculation was not performed.

## Results

### Patient characteristics

**Fig. 1:**
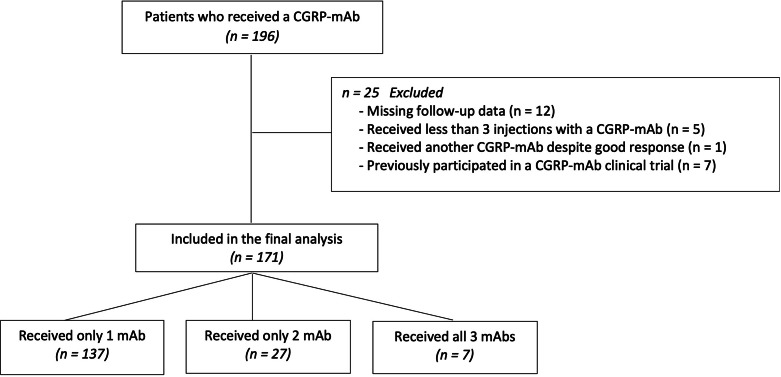
Flow chart of patient selection

In the current registry, 196 patients who received at least one mAb as preventive migraine therapy were recorded. 25 patients were excluded from the retrospective analyses due to either receiving less than 3 injections with a CGRP-mAb, missing follow-up data, switching mAb despite good response to the first CGRP-mAb or participating in a CGRP-mAb clinical trial (Fig. 1). The final sample size consisted of 171 patients, of which the majority (n = 143; 83.6%) were female. The mean age was 43.16 ± 12.54 and the distribution between EM and CM was almost even (49.7% vs. 50.3%). A history of aura was reported in one-third (32.2%) of the patients. An additional diagnosis of another headache disorder was present in 44 (25.7%) patients. Thereof, 39 (88.6%) fulfilled the diagnostic criteria for episodic TTH, 3 (6.8%) were diagnosed with another primary headache disorder than TTH. Only 2 (4.5%) patients reported a secondary headache in their past medical history. One patient was previously diagnosed with idiopathic intracranial hypertension and one with a secondary headache attributed to trauma to the head. Baseline MMD in the EM group was 10.00 [4] and in the CM group 20.00 [11]. However, 19 (22.1%) of the patients experiencing CM reported daily migraine headache. More than half (52.0%) of the study population received erenumab as their first mAb, as this product was the first to be approved in Austria. For detailed patient characteristics see Table 1.

**Table 1:** Characteristics of the study population prior to receiving the first monoclonal antibody

Characteristic	Total (n = 171)
Female, n (%)	143 (83.6)
Age in years, mean (SD)	43.16 ± 12.54
Diagnosis	
Episodic Migraine, n (%)	85 (49.7)
Chronic Migraine, n (%)	86 (50.3)
Aura, n (%)	55 (32.2)
1st MAb	
Erenumab, n (%)	89 (52.0)
Galcanezumab, n (%)	39 (22.8)
Fremanezumab, n (%)	43 (25.1)
Withdrawal, n (%)	26 (15.2)
MRM, n (%)	33 (19.3)
MOH, n (%)	62 (36.3)
Other headache disorders, n (%)	44 (25.7)
Number of prophylactic medications, median [IQR]	2.00 [2]
Non-daily migraine headache in CM, n (%)	67 (77.9)
Mean migraine days/month (EM), median	10.00 [4]
Mean migraine days/month (CM), median	20.00 [11]
Age at Migraine Diagnosis, median [IQR]	15.00 [13]
Disease duration, median [IQR]	21.00 [21]
Psychiatric comorbidity, n (%)	59 (34.5)

Mab: monoclonal antibody; MRM: menstrually related migraine; MOH: medication overuse headache;

IQR: interquartile range

### Prior preventive medication

Of the 171 patients, 147 (86.0%) had taken at least one previous preventive migraine medication, whereas 24 (14.0%) received a mAb as the first prophylactic treatment. Mean number of previously used prophylactic medication was 2.00 [2]. The four most used preventive medications included beta-blockers (n = 84; 49.1%), topiramate (n = 84; 49.1%), amitriptyline (n = 64; 37.4%) and flunarizine (n = 62;36.3%), respectively. The non-responder rate was ≧ 50% for all drugs except for topiramate (41.7%) and flunarizine (46.8%). Side effects were most frequently reported during treatment with topiramate (42.9%), followed by flunarizine (33.9%). In contrast, beta-blockers, amitriptyline as well as onabotulinumtoxinA seemed to be well tolerated with a side effect rate of less than 25% (Table 2).

**Table 2:** Previously used preventive medication before starting a monoclonal antibody including the reason for discontinuing the treatment

	Non-Responder	Side Effects	Loss-of-Efficacy	Other	Total
Beta-Blocker	48 (57.1)	18 (21.4)	7 (8.3)	11 (13.1)	84 (49.1)
Topiramate	35 (41.7)	36 (42.9)	5 (6.0)	8 (9.5)	84 (49.1)
Flunarizine	29 (46.8)	21 (33.9)	3 (4.8)	9 (14.5)	62 (36.3)
Amitriptyline	38 (59.4)	15 (23.4)	1 (1.6)	10 (15.6)	64 (37.4)
OnabotulinumtoxinA	17 (85.0)	1 (5.0)	-	2 (10.0)	20 (11.7)
Valproic acid	10 (55.6)	5 (27.8)	2 (11.1)	1 (5.6)	18 (10.5)
Candesartan	5 (62.5)	-	-	3 (37.5)	8 (4.7)
Venlafaxine	4 (57.1)	0 (0.0)	-	3 (42.9)	7 (4.1)
Tanacetum parthenium	6 (85.7)	-	-	1 (14.3)	7 (4.1)
Other	23 (65.7)	4 (11.4)	-	8 (22.9)	35 (20.5)

Values are absolute numbers and percent (%)

### CGRP non-responders and loss-of-efficacy

**Fig. 2:**
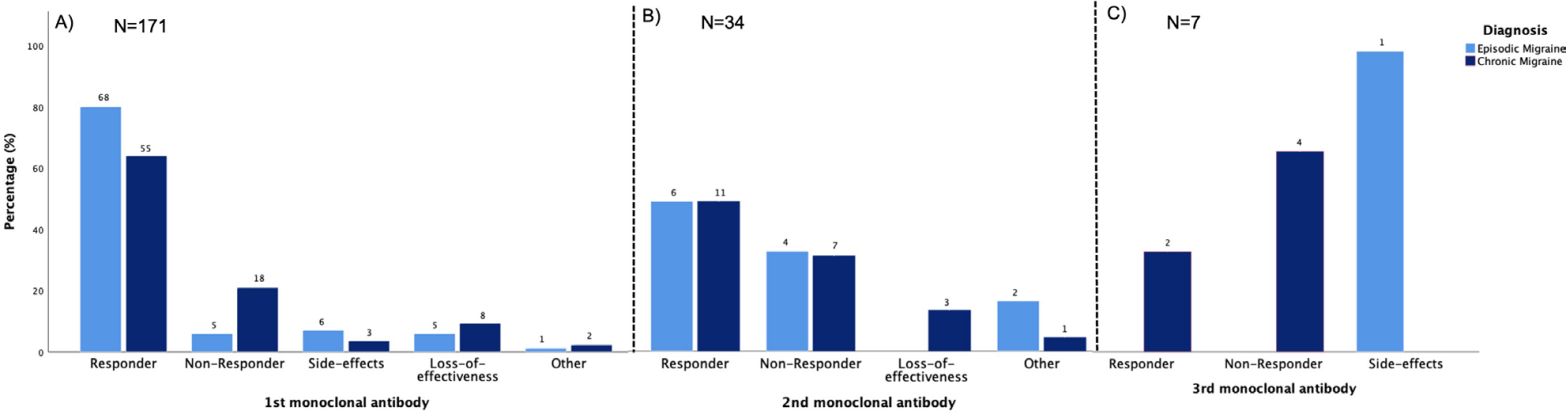
Treatment response for patients diagnosed with EM or CM receiving the first (N = 171), second (N = 34) or third (N = 7) monoclonal antibody. Absolute numbers are given; percentages can be gathered from the y-axis. Response was defined as ≥ 50% reduction of monthly migraine days (MMDs) in episodic migraine and ≥ 30% reduction of MMDs in chronic migraine after an adequate treatment duration of at least 3 months. Vice versa, non-response was defined as ≤ 50% reduction of MMDs in EM and ≤ 30% reduction of MMDs in CM after 3 months of treatment. Loss of effectiveness was defined as an initial response, but with a subsequent increase in migraine frequency back to or beyond the baseline level. Side effects that led to a discontinuation or switch of the treatment were rare and included erythema, itching and swelling at the site of injection, muscle cramps as well as constipation. Other reasons for stopping/switching the treatment included for example reimbursement issues

Of the 171 patients included in the present analysis, 137 (80.1%) received only one mAb, 27 (15.8%) received only two mAbs and 7 (4.1%) were treated with all three mAbs (see Fig. 2). The overall response rate to the first monoclonal antibody was 71.9%. However, patients with EM were more likely to respond to the treatment (80.0% vs.64.0%, p = 0.03). 23 (13.5%) did not respond to the treatment with their first monoclonal antibody. Of those, 5 patients had been diagnosed with EM and 18 patients with CM. 13 (7.6%) experienced a loss of effectiveness during the treatment with the first mAb, which occurred after a mean of 8.00 ± 6.78 months of therapy, respectively. 11 (84.6%) patients, who experienced a loss of efficacy, received erenumab as their first antibody, one (7.7%) galcanezumab and one (7.7%) fremanezumab. Side effects were rare and led to discontinuation of therapy in 5.3% of the cases (Fig. 2, A). Adverse events included erythema (n = 2), itching, and swelling at the site of injection (n = 2), muscle cramps (n = 1) as well as constipation (n = 3). An allergic reaction was suspected in one patient – however, a causal association could not be verified.

**Fig. 3:**
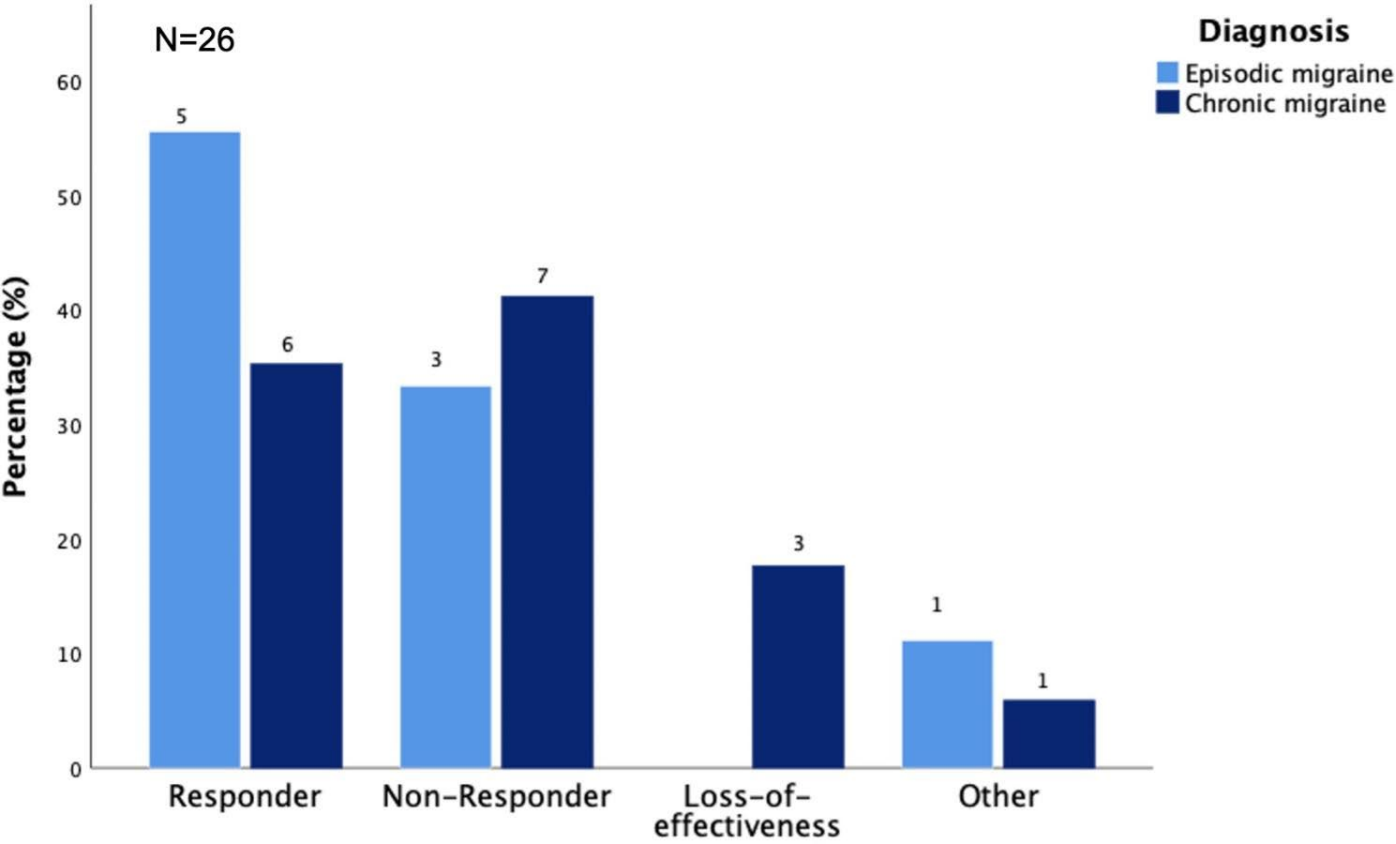
Responder rates for patients with EM or CM who switched mAb-class and received a second CGRP-antibody due to lack or loss of effectiveness of the first monoclonal antibody (N = 26). Absolute numbers are given; percentages can be gathered from the y-axis. Patients who were first treated with a CGRP-receptor received a CGRP-ligand as second drug and vice versa

17 (50%) of the 34 patients, who received a second antibody, responded to the mAb-class switch (6 were diagnosed with EM, 11 were diagnosed with CM; see Fig. 2, B). Considering only those who did not respond to or experienced a loss of efficacy to the first antibody (N = 26; EM = 9; CM = 17), 11 (42.3%) responded to the second monoclonal antibody whereas 10 (38.5%) did not benefit from an antibody switch. 5 (19.2%) experienced a loss of efficacy or discontinued the treatment due to other reasons (Fig. 3). Loss of efficacy occurred after 3 months, respectively.

Altogether, 7 patients received all 3 mAbs. Two of them responded to the third mAb (both diagnosed with CM, one with daily migraine headache) despite not responding to the first two CGRP-mAb treatments.

Non-response to a monoclonal antibody was associated with a higher monthly migraine frequency (p = 0.028) and loss of efficacy was associated with medication overuse (p = 0.022). Both were linked to a higher number of prior preventive migraine therapies (p = 0.011 and p = 0.022, respectively) (Table 3). Daily migraine attacks did not affect response.

**Table 3:** Associated risk factors for non-response or loss-of-effectiveness in patients who received their first CGRP(R)-monoclonal antibody

	Non-Responder (N = 23)				Loss-of-Efficacy (N = 13)		
	OR	CI (95%)	Sig.		OR	CI (95%)	Sig.
Female Sex	0.582	(0.13.-2.60)	0.479		0.46	(0.06–3.41)	0.445
Age	0.98	(0.93–1.02)	0.267		0.94	(0.88–1.01)	0.051
MMD	**1.09**	**(1.01–1.17)**	**0.028**		1.41	(1.25–1.60)	0.496
Psychiatric disorder	2.90	(0.38–8.53)	0.055		6.05	(0.99–36.81)	0.051
Medication overuse	1.21	(0.21–3.61)	0.749		**7.60**	**(1.33–43.45)**	**0.022**
Withdrawal	0.88	(2.27–5.16)	0.853		0.70	(0.23–8.67)	0.406
Number of prophylactic medications	**1.08**	**(1.11–2.06)**	**0.011**		**1.59**	**(1.07–2.35)**	**0.022**

Results for multinomial logistic regression models with the Response to the first monoclonal antibody as dependent variable

CI: confidence interval; MMD: monthly migraine days; OR: odds ratio

Bold numbers indicate that coefficients are statistically significant

## Discussion

This is the first study investigating treatment response in refractory migraine patients who underwent preventive therapy with up to 3 different monoclonal antibodies. Our observations confirm the benefit of a mAb-class switch if treatment with the first antibody failed due to lack or loss of efficacy. Therefore, we postulate that an antibody switch results in an adequate therapeutic response in a subset of migraineurs.

Recommendations of international headache societies as the EHF or the American Headache Society (AHS) include offering CGRP-mAbs to patients with EM or CM who were unable to tolerate or showed an inadequate response to two of the evidence-based preventive treatments such as topiramate, beta-blockers or flunarizine [25–27]. However, in practice, the use of CGRP-mAbs is restricted and reflects different reimbursement conditions in several countries. In Germany, for example, the prerequisite for reimbursement of treatment with galcanezumab or fremanezumab is 4 (in EM) to 5 (in CM) failed migraine preventives, whereas erenumab can be prescribed after only one treatment failure.

In Austria however, only 3 preceding migraine therapies are required to qualify for treatment with a mAb. Besides, despite growing evidence on the positive effect of an antibody switch in non-responders to a different CGRP-mAb treatment [20], in some countries, treatment with only one CGRP-mAb is possible from a reimbursement perspective.

Our analysis indicates that an increasing number of prior preventive therapies are associated with lower efficacy in treatment response to mAbs. However, we are aware of the fact that especially patients with a long migraine history and/or chronic migraine, have often tried a variety of preventive migraine therapies – it is particularly this patient population which is rather difficult to treat [28].

Trials including patients with 2–4 prior migraine preventive treatment failures showed a ≥ 50%-response of 38.4% for galcanezumab, of 30% for erenumab and 34% for fremanezumab [29–31]. Considering only those patients with at least two prior therapies, the response rate in our analysis is 67.6%. Our response rates are thus higher, but comparable to other real-world data [14]. However, it should be noted that we have determined a ≥ 30% response rate in chronic migraine patients. In the current analysis, 24 patients without a history of prior migraine preventive therapies were included. These patients had contraindications for the standard-of-care (SOC) preventive migraine therapies. The response rate for this cohort was higher than in those with ≥ 3 prior treatments but failed statistical significance. Based on our observations and concerning the favourable tolerability, an earlier use of CGRP-mAbs in the prevention of migraine could be considered. However, only long-term data will reveal the effectiveness of monoclonal antibodies targeting the CGRP pathway in preventing migraine chronification.

Furthermore, the analysis showed that of 171 patients only 5.3% discontinued mAb therapy due to side effects, which were mainly redness, itching and swelling at the site of injection as well as constipation.

Whether therapy with an CGRP-mAb leads to fewer side effects than the previous SOC migraine preventive treatments is currently subject of research. A recently published head-to-head study compared the tolerability and efficacy of erenumab (70 mg or 140 mg monthly s.c.) to topiramate (100 mg daily) [18] and indicated that 10.6% of patients with erenumab but 38.9% in the topiramate group discontinued therapy due to adverse drug reactions. Further studies comparing preventive therapy with standard oral medications and with CGRP-mAbs are ongoing. Moreover, a lower rate of side effects with CGRP-mAb therapy compared with SOC medication would support the earlier use of CGRP-mAbs in migraine therapy.

The reason why patients who previously did not respond to therapy with the initial CGRP-mAb, show a therapeutic response to treatment with a second or a third mAb is still elusive. Besides blocking different CGRP pathways (i.e. receptor versus ligand) an additional explanation might be the different immunglobuline G (IgG) subclasses of the available antibodies. Currently, there is limited data available regarding the effect of IgG subclass on pharmacokinetics and pharmacodynamics of monoclonal antibodies [32]. Furthermore, the antibodies bind with different affinity and specificity to CGRP and closely related calcitonin family members [33].

### Limitations

The main limitation of the study is the retrospective study design and the small number of patients who received two or three mAbs. However, the sample size is comparable to the previous study investigating effects of CGRP-mAb switch [20]. The dataset is restricted to the information of the structured face-to-face headache interview and medical report taken during the patients’ visit to our headache outpatient clinic. However, as this is an open case series and patients were continuously enrolled from April 2018 until December 2021, the observational period for each patient varies. Follow-up visits were not strictly carried out after a particular time period but rather in a time frame between 3 and 12 months after treatment initiation with a mAb reflecting real world practice.

Inherent to retrospective design, selection bias, recall bias and regression to the mean bias cannot be fully excluded.

## Conclusion

Our retrospective analysis describes a therapeutic approach using a mAb-class switch if treatment with the first antibody failed to reduce migraine frequency. In addition, we were able to show for the first time, that a therapeutic approach with a third CGRP-mAb might be a possible treatment option to achieve adequate response. As a high number of previously ineffective preventive migraine therapies are associated with a negative response, earlier use of CGRP-mAbs for patients might be appropriate. Prospective studies are needed to confirm the benefit of switching CGRP®mAb in migraine patients.

## Data Availability

All data generated or analysed during this study are included in this published article.

## Abbreviations

AHS: American Headache Society
CGRP: Calcitonin gene related peptide
CGRP(R): Calcitonin gene related peptide – receptor
CM: Chronic migraine
EHF: European Headache Federation
EM: Episodic migraine
ICHD-3: International Classification of Headache Disorders, 3rd edition
IQR: Inter quartile range
mAB: Monoclonal antibody
MMD: Monthly migraine days
MOH: Medication-overuse-headache
MO: Medication-overuse
NSAID: Non-steroidal anti-inflammatory drug
SD: Standard deviation
TAC: Trigeminal-autonomic cephalalgia
TTH: Tension-type headache
